# An absorption-free and Doppler-improved optical waveguide for diffractionless light propagation

**DOI:** 10.1038/s41598-017-14456-z

**Published:** 2017-10-27

**Authors:** Ni Cui, Ziyang Gan, Lida Zhang

**Affiliations:** 1 Siyuan Laboratory, Guangzhou Key Laboratory of Vacuum Coating Technologies and New Energy Materials, Department of Physics, Jinan University, Guangzhou, 510632 China; 2 Guangdong Provincial Key Laboratory of Optical Fiber Sensing and Communications, Jinan University, Guangzhou, 510632 China; 3 Texas A & M University, College Station, Texas 77843 United States

## Abstract

We propose a novel scheme to realize an optical waveguide induced by an active Raman gain (ARG) process in a four-level *N*-type atomic system. Because of the nature of the ARG, there are two distinct features related to the waveguide: i) It is not absorptive, on the contrary, weak gain is presented; ii) It can be improved by the Doppler effect in the sense that the dispersion is enhanced while the gain is further reduced. This is in sharp contrast to the previously considered schemes where usually the optical induced waveguide is passive and is severely attenuated by the Doppler effect. We then study the paraxial light propagation in the waveguide which shows that the propagation dynamics is lossless and diffractionless.

## Introduction

Optical diffraction, originating from the wave nature of light, is inevitable during beam propagation. It usually leads to power attenuation, width broadening, image distortion, and is also deleterious to spatial squeezing in the quantum regime^1,2^. In order to overcome the diffraction, a plenty of methods has been developed in the last years. Maybe the most common way is to employ an optical fiber which is designed to have low-high-low refractive index contrast and is capable of guiding light in a confined region^3^. And it is also known that there are specific characteristic spatial modes which are immune to diffraction^4–11^. These methods are of great importance in optical-related technologies while might be subject to the lack of flexibility in the sense that they are either not tunable or may only work for certain spatial modes.

An alternative method which provides additional flexibility is to utilize light-atom interaction to reduce diffraction. The physical essence is to couple strong spatial-dependent control laser beams to atomic systems such that its linear susceptibility is accordingly tailored, forming an optically induced waveguide for a weak probe beam. This optically induced waveguide is tunable owing to the fact that it crucially depends on the spatial structure of the laser fields which can be easily manipulated. In this context, many interesting theoretical schemes have been proposed based on atomic coherence effects such as electromagnetically induced transparency^12–17^, coherent population trapping^18–20^, and also have been experimentally verified^21–23^. Apart from the physical mechanism based on structured control beam, another interesting approach is to explore atomic motions and collision to cancel paraxial diffraction^24–27^. In most of these proposals, the strong control and weak probe beams are coupled to the atomic transitions in the near-resonance regime. This may be subject to two drawbacks: (i) In the near-resonance regime, the probe beam propagates without diffraction owing to the induced waveguide but often accompanied with strong absorption, leading to a severely attenuated output. (ii) The atomic response will be usually weakened by the Doppler effect, making it hard and impractical to work at room temperature.

Particular interest has been devoted to three-level lambda Raman systems where a gain is presented^21,28^. Furthermore, it has been recently realized that the linear response can be enhanced and narrowed by the Doppler effect in a four-level N-type atomic system where an active Raman gain process is formed^29^. This scheme has been also further explored to strengthen the precursor effect at room temperature^30^. Based on this paper, we find that this system can be used to realize lossless and diffractionless propagation under proper conditions, overcoming the two disadvantages as mentioned earlier. This paper is organized as follows: Firstly, we present the theoretical model for the four-level atomic system. Then, we discuss how to induce the optical waveguide and show that it can be improved by the Doppler effect in the sense that the linear gain is reduced. In the next section, we study the beam propagation dynamics, showing that lossless and diffractionless propagation can be achieved. In the end, we present a brief discussion and summary for our work.

## Theoretical Model

**Figure 1:**
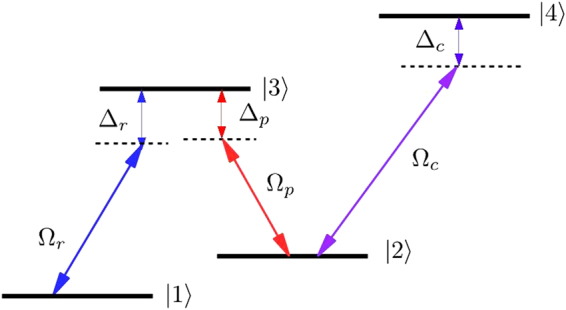
The four-level *N*-type Scheme consisting of two lower states |1〉 and |2〉, two upper states |3〉 and |4〉. The probe field Ω_*p*_ couples to transition |3〉 ↔ |2〉, while the other transitions |3〉 ↔ |1〉 and |4〉 ↔ |2〉 are driven by two coherent control fields with a Rabi frequencies Ω_*r*_ and Ω_*c*_, respectively.

Here, we consider an *N*-type four-level atomic system, the energy levels are shown in Fig. 1. The transitions |3〉 ↔ |1〉 and |3〉 ↔ |2〉 are coupled by the Raman and probe fields with Rabi frequencies Ω_*r*_ and Ω_*p*_, respectively, while the transition |4〉 ↔ |2〉 is driven by a control field with Rabi frequency Ω_*c*_. As we show below, the Raman field Ω_*r*_ is chosen to be spatially dependent field such that an optical wave guide is induced for the probe field.

In the dipole and rotating-wave approximation, one can obtain the interaction Hamiltonian of the system as

1 $$\begin{array}{c}{H}_{I}=-\hslash ({{\rm{\Delta }}}_{r}-{{\rm{\Delta }}}_{p}\mathrm{)|2}\rangle \langle \mathrm{2|}-\hslash {{\rm{\Delta }}}_{r}\mathrm{|3}\rangle \langle \mathrm{3|}-\hslash ({{\rm{\Delta }}}_{r}-{{\rm{\Delta }}}_{p}+{{\rm{\Delta }}}_{c}\mathrm{)|4}\rangle \langle \mathrm{4|}\\ \,\,\,\,\,\,\,\,\,\,\,\,-\hslash ({{\rm{\Omega }}}_{r}\mathrm{|1}\rangle \langle \mathrm{3|}+{{\rm{\Omega }}}_{p}\mathrm{|2}\rangle \langle \mathrm{3|}+{{\rm{\Omega }}}_{c}\mathrm{|2}\rangle \langle \mathrm{4|}+{\rm{H}}{\rm{.c}}\mathrm{.),}\end{array}$$

where ${{\rm{\Delta }}}_{r}={\omega }_{r}-{\omega }_{31}$, ${{\rm{\Delta }}}_{p}={\omega }_{p}-{\omega }_{32}$, ${{\rm{\Delta }}}_{c}={\omega }_{c}-{\omega }_{42}$ are the detunings of the laser fields, and the Rabi frequencies of the fields are defined as ${{\rm{\Omega }}}_{r}={\overrightarrow{d}}_{31}\cdot {\overrightarrow{E}}_{r}/\mathrm{(2}\hslash )$, ${{\rm{\Omega }}}_{p}={\overrightarrow{d}}_{32}\cdot {\overrightarrow{E}}_{p}/\mathrm{(2}\hslash )$ and ${{\rm{\Omega }}}_{c}={\overrightarrow{d}}_{42}\cdot {\overrightarrow{E}}_{c}/\mathrm{(2}\hslash )$, respectively. Here, ${\overrightarrow{d}}_{ij}((i,j)\in \mathrm{\{1,2,3,4\})}$ are the dipole moments of the respective transitions $|i\rangle \leftrightarrow |j\rangle$. The master equation of motion follows as

2 $$\dot{\rho }=-\frac{i}{\hslash }[{H}_{I},\rho ]- {\mathcal L} \rho ,$$

and ${\mathcal L}$ *ρ* represents the incoherent relaxation processes, which is determined by

3a $$ {\mathcal L} \rho ={ {\mathcal L} }_{31}^{\gamma }\rho +{ {\mathcal L} }_{32}^{\gamma }\rho +{ {\mathcal L} }_{41}^{\gamma }\rho +{ {\mathcal L} }_{42}^{\gamma }\rho +{ {\mathcal L} }_{21}^{\gamma }\rho +{ {\mathcal L} }^{d}\rho ,$$

3b $${ {\mathcal L} }_{jk}^{\gamma }=\frac{{{\rm{\Gamma }}}_{jk}}{2}(|j\rangle \langle j|\rho +\rho |j\rangle \langle j|-\mathrm{2|}k\rangle \langle j|\rho |j\rangle \langle k|),$$

3c $${ {\mathcal L} }^{d}=\sum _{j\ne k}{\gamma }_{jk}^{d}|j\rangle \langle k|,$$

here, ${{\rm{\Gamma }}}_{jk}$ describes the spontaneous decay rate from |*j*〉 to |*k*〉, and ${\gamma }_{ij}^{d}$ being the pure dephasing rate for ${\rho }_{ij}$. The equations of motion for the relevant density matrix elements can easily be derived as

4a $${\dot{\rho }}_{11}=-i{{\rm{\Omega }}}_{r}({\rho }_{13}-{\rho }_{31})+{{\rm{\Gamma }}}_{31}{\rho }_{33}+{{\rm{\Gamma }}}_{41}{\rho }_{44},$$

4b $${\dot{\rho }}_{22}=-i{{\rm{\Omega }}}_{c}({\rho }_{24}-{\rho }_{42})-i{{\rm{\Omega }}}_{p}({\rho }_{23}-{\rho }_{32})+{{\rm{\Gamma }}}_{32}{\rho }_{33}+{{\rm{\Gamma }}}_{42}{\rho }_{44}-{{\rm{\Gamma }}}_{21}{\rho }_{22},$$

4c $${\dot{\rho }}_{33}=i{{\rm{\Omega }}}_{r}({\rho }_{13}-{\rho }_{31})+i{{\rm{\Omega }}}_{p}({\rho }_{23}-{\rho }_{32})-({{\rm{\Gamma }}}_{31}+{{\rm{\Gamma }}}_{32}){\rho }_{33},$$

4d $${\dot{\rho }}_{21}=-({\gamma }_{21}+i({{\rm{\Delta }}}_{p}-{{\rm{\Delta }}}_{r})){\rho }_{21}-i({{\rm{\Omega }}}_{r}{\rho }_{23}-{{\rm{\Omega }}}_{p}{\rho }_{31}-{{\rm{\Omega }}}_{c}{\rho }_{41}),$$

4e $${\dot{\rho }}_{31}=-({\gamma }_{31}-i{{\rm{\Delta }}}_{r}){\rho }_{31}+i({{\rm{\Omega }}}_{p}{\rho }_{21}+{{\rm{\Omega }}}_{r}({\rho }_{11}-{\rho }_{33})),$$

4f $${\dot{\rho }}_{32}=-({\gamma }_{32}-i{{\rm{\Delta }}}_{p}){\rho }_{32}+i({{\rm{\Omega }}}_{r}{\rho }_{12}-{{\rm{\Omega }}}_{c}{\rho }_{34}+{{\rm{\Omega }}}_{p}({\rho }_{22}-{\rho }_{33})),$$

4g $${\dot{\rho }}_{41}=-({\gamma }_{41}-i({{\rm{\Delta }}}_{p}-{{\rm{\Delta }}}_{r}-{{\rm{\Delta }}}_{c})){\rho }_{41}+i({{\rm{\Omega }}}_{c}{\rho }_{21}-{{\rm{\Omega }}}_{r}{\rho }_{43}),$$

4h $${\dot{\rho }}_{42}=-({\gamma }_{42}-i{{\rm{\Delta }}}_{c}){\rho }_{42}+i({{\rm{\Omega }}}_{c}({\rho }_{22}-{\rho }_{44})-{{\rm{\Omega }}}_{p}{\rho }_{43}),$$

4i $${\dot{\rho }}_{43}=-({\gamma }_{43}-i({{\rm{\Delta }}}_{c}-{{\rm{\Delta }}}_{p})){\rho }_{43}+i({{\rm{\Omega }}}_{c}{\rho }_{23}-{{\rm{\Omega }}}_{p}{\rho }_{42}-{{\rm{\Omega }}}_{r}{\rho }_{41})\mathrm{.}$$

Here, the remaining equations follow the contraints ${\sum }_{i}{\rho }_{ii}=1$ and ${\rho }_{ij}={\rho }_{ji}^{\ast }$, with $(i,j)\in \mathrm{\{1,2,3,4\}}$. ${\gamma }_{ij}={\gamma }_{ij}^{d}+$ $({{\rm{\Gamma }}}_{i}+{{\rm{\Gamma }}}_{j}\mathrm{)/2}$ is the total damping rate, and ${{\rm{\Gamma }}}_{j}={\sum }_{k}{{\rm{\Gamma }}}_{jk}$ being the total decay rate out of the state $|j\rangle$.

In the following, we consider the far detuned regime for the probe and Raman fields where ${{\rm{\Omega }}}_{r},{{\rm{\Omega }}}_{c},{{\rm{\Delta }}}_{c},{\gamma }_{ij}\ll {{\rm{\Delta }}}_{p},{{\rm{\Delta }}}_{r}$ and also the weak probe limit when ${{\rm{\Omega }}}_{p}\ll {{\rm{\Omega }}}_{r},{{\rm{\Omega }}}_{c}$. By further choosing the near two-photon resonant condition ${{\rm{\Delta }}}_{p}\sim {{\rm{\Delta }}}_{r}$, the Ω_*p*_ and Ω_*r*_ will form a Raman process. Because of the two-photon Raman process in the sub-lambda system, there will be some population in the state |2〉. However, it will be driven to state |4〉 by the pump effect of the control field Ω_*c*_ and then decay back to the state |1〉 by spontaneous emission. Therefore, almost all of the atoms would be in ground state |1〉, and the probe field will experience a weak gain but no absorption owing to the active Raman process. This is one of the two most important features in our system that differs from previous studies which were usually operated in a passive system when loss is inevitable. In these conditions, the zeroth-order density matrix elements for the probe field can be calculated as

5a $${\rho }_{11}^{\mathrm{(0)}}\simeq \mathrm{1,}$$

5b $${\rho }_{13}^{\mathrm{(0)}}={{\rm{\Omega }}}_{r}{\rho }_{11}^{\mathrm{(0)}}/(-{{\rm{\Delta }}}_{r}+i{\gamma }_{13})\mathrm{.}$$

Here, only the nonzero elements are shown above. Then, the first-order element ${\rho }_{32}^{\mathrm{(1)}}$ corresponding to the linear medium response for the probe field, can be obtained as

6 $${\rho }_{32}^{\mathrm{(1)}}=\frac{A{{\rm{\Omega }}}_{p}{\rho }_{13}^{\mathrm{(0)}}}{B}\mathrm{.}$$

The expressions for *A*, *B* are given as

7a $$A={{\rm{\Omega }}}_{r}({{\rm{\Omega }}}_{c}^{2}-{{\rm{\Omega }}}_{r}^{2}+({{\rm{\Delta }}}_{p}-{{\rm{\Delta }}}_{r}-{{\rm{\Delta }}}_{c}+i{\gamma }_{14})({{\rm{\Delta }}}_{p}-{{\rm{\Delta }}}_{c}+i{\gamma }_{34})),$$

7b $$\begin{array}{c}B=({{\rm{\Delta }}}_{p}-{{\rm{\Delta }}}_{r}+i{\gamma }_{12})({{\rm{\Delta }}}_{p}+i{\gamma }_{32})({{\rm{\Delta }}}_{p}-{{\rm{\Delta }}}_{r}-{{\rm{\Delta }}}_{c}+i{\gamma }_{14})\\ \,\,\,\,\,\,\,\,({{\rm{\Delta }}}_{p}-{{\rm{\Delta }}}_{c}+i{\gamma }_{34})-(({{\rm{\Delta }}}_{p}-{{\rm{\Delta }}}_{r}+i{\gamma }_{12})({{\rm{\Delta }}}_{p}-{{\rm{\Delta }}}_{r}-{{\rm{\Delta }}}_{c}+i{\gamma }_{14})+({{\rm{\Delta }}}_{p}+i{\gamma }_{32})({{\rm{\Delta }}}_{p}-{{\rm{\Delta }}}_{c}+i{\gamma }_{34})){{\rm{\Omega }}}_{c}^{2}-(({{\rm{\Delta }}}_{p}-{{\rm{\Delta }}}_{r}+i{\gamma }_{12})({{\rm{\Delta }}}_{p}+i{\gamma }_{32})\\ +({{\rm{\Delta }}}_{p}-{{\rm{\Delta }}}_{r}-{{\rm{\Delta }}}_{c}+i{\gamma }_{14})({{\rm{\Delta }}}_{p}-{{\rm{\Delta }}}_{c}+i{\gamma }_{34})){{\rm{\Omega }}}_{r}^{2}\\ +{({{\rm{\Omega }}}_{c}^{2}-{{\rm{\Omega }}}_{r}^{2})}^{2}\mathrm{.}\end{array}$$

From Eq. (6), the linear susceptibility of the probe field can be written as

8 $$\chi =\frac{3N{\lambda }_{p}^{3}}{8{\pi }^{2}}\frac{{{\rm{\Gamma }}}_{32}{\rho }_{32}^{\mathrm{(1)}}}{{{\rm{\Omega }}}_{p}}=\frac{3N{\lambda }_{p}^{3}}{8{\pi }^{2}}\frac{A{{\rm{\Gamma }}}_{32}{{\rm{\Omega }}}_{r}}{B(-{{\rm{\Delta }}}_{r}+i{\gamma }_{13})},$$

where *N* is the atomic density and *λ*_*p*_ is the wavelength of the probe field.

The propagation dynamics of the probe field is governed by the Maxwell’s wave equations. In the slowly varying envelope approximation and the paraxial wave approximation, the motion equation for the probe field propagation along the *z* direction can be derived as

9 $$\frac{\partial {E}_{p}}{\partial z}=\frac{i}{2{k}_{p}}{\nabla }_{\perp }^{2}{E}_{p}+\frac{i{k}_{p}}{2}\chi {E}_{p},$$

where ${k}_{p}={\omega }_{p}/c$, with *c* being the speed of light in vacuum, and ${\nabla }_{\perp }^{2}={\partial }^{2}/\partial {x}^{2}+{\partial }^{2}/\partial {y}^{2}$ which introduces paraxial diffraction for the probe. As we have discussed earlier, paraxial diffraction would lead to width broadening, energy spreading, and image distortion for the propagating laser beam, which is usually detrimental and required to be removed for most applications such as optical communications. In the next section, we will show how to modify the linear susceptibility by controlling the spatial structure of the Raman field to form an active waveguide-like structure. As a result, the probe field will propagate in the medium without diffraction. In particular, in this optically induced waveguide, the probe will experience no absorption because of the nature of the active Raman process.

## Optically Induced Waveguide-Like Structure

### Absorption-free optical waveguide

Though the linear susceptibility in the ARG system has already been studied in different contexts^29,30^, it would be useful to firstly investigate *χ* in order to find the optimal condition for the creation of the desired waveguide structure in our ARG system. An ideal waveguide would assume a large refractive index contrast with no absorption or gain. To create the optical waveguide as good as possible, it is obvious that the following criteria should be satisfied

10a $${\rm{Re}}[\chi ] > 0\,,$$

10b $${\rm{Abs}}[\frac{{\rm{Re}}[\chi ]}{{\rm{Im}}[\chi ]}]\gg 1,$$

where the first condition essentially assures that the refractive index is positive such that a waveguide is possible, while the second one means that the loss or amplification caused by ${\rm{Im}}[\chi ]$ can be neglected during the propagation of the probe field. This suggests to us that the conditions for maximal ${\rm{Re}}[\chi ]$ which is usually accompanied by a strong absorption/gain may not be optimal for the waveguide.

**Figure 2:**
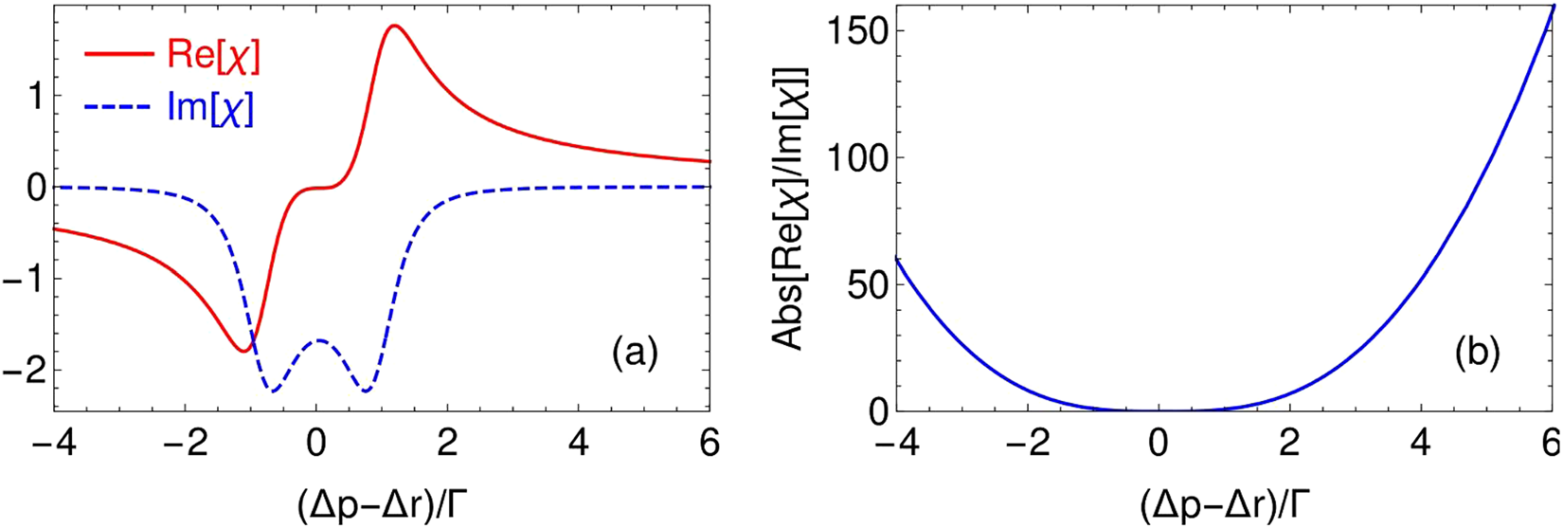
(**a**) The linear dispersion ${\rm{Re}}[\chi ]$ and absorption ${\rm{Im}}[\chi ]$ as a function of probe detuning. It is obvious that the absorption is always below zero due to the nature of the ARG process. We then plot ${\rm{Abs}}[{\rm{Re}}[\chi ]/{\rm{Im}}[\chi ]]$ in (**b**) which indicates that the optimal condition for the waveguide structure should be found in the region ${{\rm{\Delta }}}_{p} > {{\rm{\Delta }}}_{p0}$. Parameters are chosen as $N=1.0\times {10}^{12}\,{{\rm{cm}}}^{-3}$, ${\lambda }_{p}\mathrm{=795\ }{\rm{nm}}$, ${{\rm{\Gamma }}}_{31}={{\rm{\Gamma }}}_{32}={{\rm{\Gamma }}}_{41}$ = ${{\rm{\Gamma }}}_{42}={\rm{\Gamma }}=\pi \times 6\,{\rm{MHz}}$, ${\gamma }_{31}={\gamma }_{32}={{\rm{\Gamma }}}_{32}$, ${\gamma }_{41}={\gamma }_{42}={{\rm{\Gamma }}}_{42}$, ${\gamma }_{12}\,=\,0.01{{\rm{\Gamma }}}_{32}$, ${\gamma }_{34}={{\rm{\Gamma }}}_{32}+{{\rm{\Gamma }}}_{42}$, ${{\rm{\Delta }}}_{r}=\mathrm{200\ }{\rm{\Gamma }}$, ${{\rm{\Delta }}}_{c}\,=0\,$, ${{\rm{\Omega }}}_{c}=\mathrm{1\ }{\rm{\Gamma }}$, ${{\rm{\Omega }}}_{r0}=\mathrm{3\ }{\rm{\Gamma }}$.

We then plot the linear dispersion and absorption as a function of the probe field detuning under certain Raman and control field parameters as shown in Fig. 2. As expected, the absorption is indeed negative owing to the active Raman process; moreover, by choosing the probe detuning away from the two photon resonance ${{\rm{\Delta }}}_{p0}\simeq {{\rm{\Delta }}}_{r}+{{\rm{\Omega }}}_{c}+\frac{{{\rm{\Omega }}}_{r}^{2}}{{{\rm{\Delta }}}_{r}}$, the probe field gain can be very weak such that the effect of amplification for the probe field due to Raman gain is not important for the probe field.

**Figure 3:**
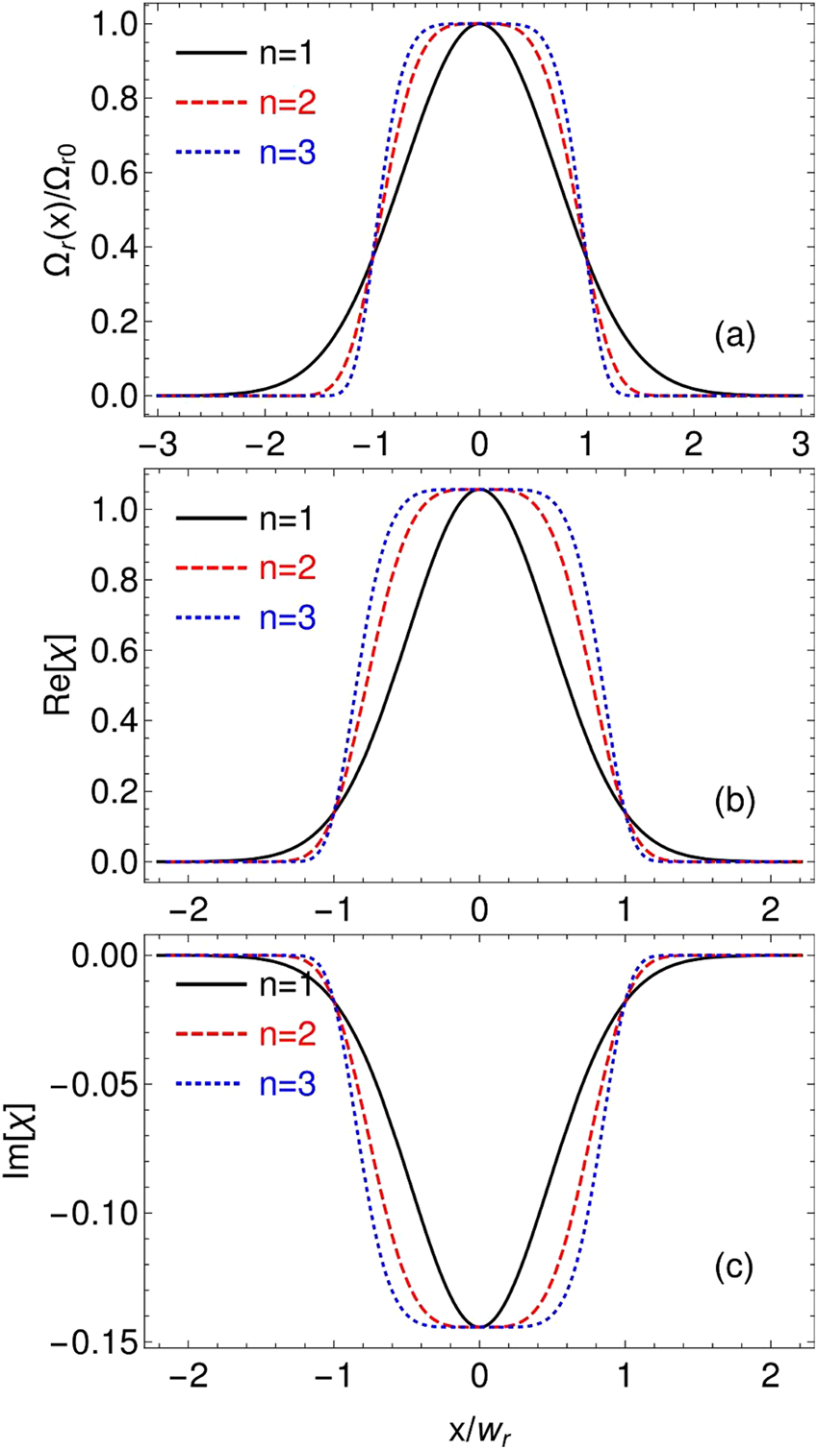
(**a**) Real and (**b**) imaginary parts of the linear susceptibility as a function of the position for different *n*. It is obvious that a better optical waveguide is formed for higher *n*. Here we choose ${{\rm{\Delta }}}_{p}=202{\rm{\Gamma }}$, other parameters are the same as in Fig. 2.

The idea to create a waveguide-like structure is based on the fact that the linear susceptibility for the probe field has a strong dependence on intensity distributions of the Raman field. By tuning the spatial properties of the Raman field in the transverse direction, one would be able to modulate the linear medium in such a way that it can guide the probe field propagation. Here, we firstly assume that the Raman field Ω_*r*_ has a one dimension (1D) super-Gaussian profile

11 $${{\rm{\Omega }}}_{r}={{\rm{\Omega }}}_{r0}{e}^{-{(\frac{{x}^{2}}{2{w}_{r}^{2}})}^{n}}\mathrm{,\ \ \ \ }n=\mathrm{1,\; 2,\; 3,...,}$$

where *w*_*r*_ is the width of the intensity profile, and *n* is the order of the super-Gaussian profile. We then plot *χ* in Fig. 3 for different n. As shown in Fig. 3(a), for $n\,=\,1$, the Raman field has a normal Gaussian profile with gradually deceasing wings. When *n* becomes larger, the two wings change to two sharply dropping edges, forming a flat-top region in the center of the field profile, which resembles a waveguide-like structure.

We then plot the real and imaginary parts of the linear susceptibility for different *n* respectively. As *n* increases, it is easily seen from Fig. 3(b) that the spatial dependence of the linear dispersion ${\rm{Re}}[\chi ]$ follows that of the Raman field; meanwhile, the linear absorption ${\rm{Im}}[\chi ]$ remains to be negative and negligible compared to the dispersion ${\rm{Re}}[\chi ]$, see the vertical scale in Fig. 3(c). In total, it means that we have optically induced a 1D absorption-free waveguide inside the atomic medium. The extension to the two-dimensional case would be straightforward.

It would be worthwhile to discuss the underlying physics resulting in better waveguide structure as *n* increases. We understood this as follows: in the dressed-state picture, the effect of the Raman field would be to introduce a space-dependent Stark shift equal to ${{\rm{\Omega }}}_{r}^{2}(x)/{{\rm{\Delta }}}_{r}$ for the probe transition. This Stark shift is proportional to the Raman field intensity (but not the amplitude), which means that the linear dispersion for the probe field depends very sensitively on the spatial distribution of the Raman field. This is apparently different from the near-resonance case where the energy shift of the dressed states varies linearly with the amplitude of the control field.

### Doppler-improved optical waveguide

**Figure 4:**
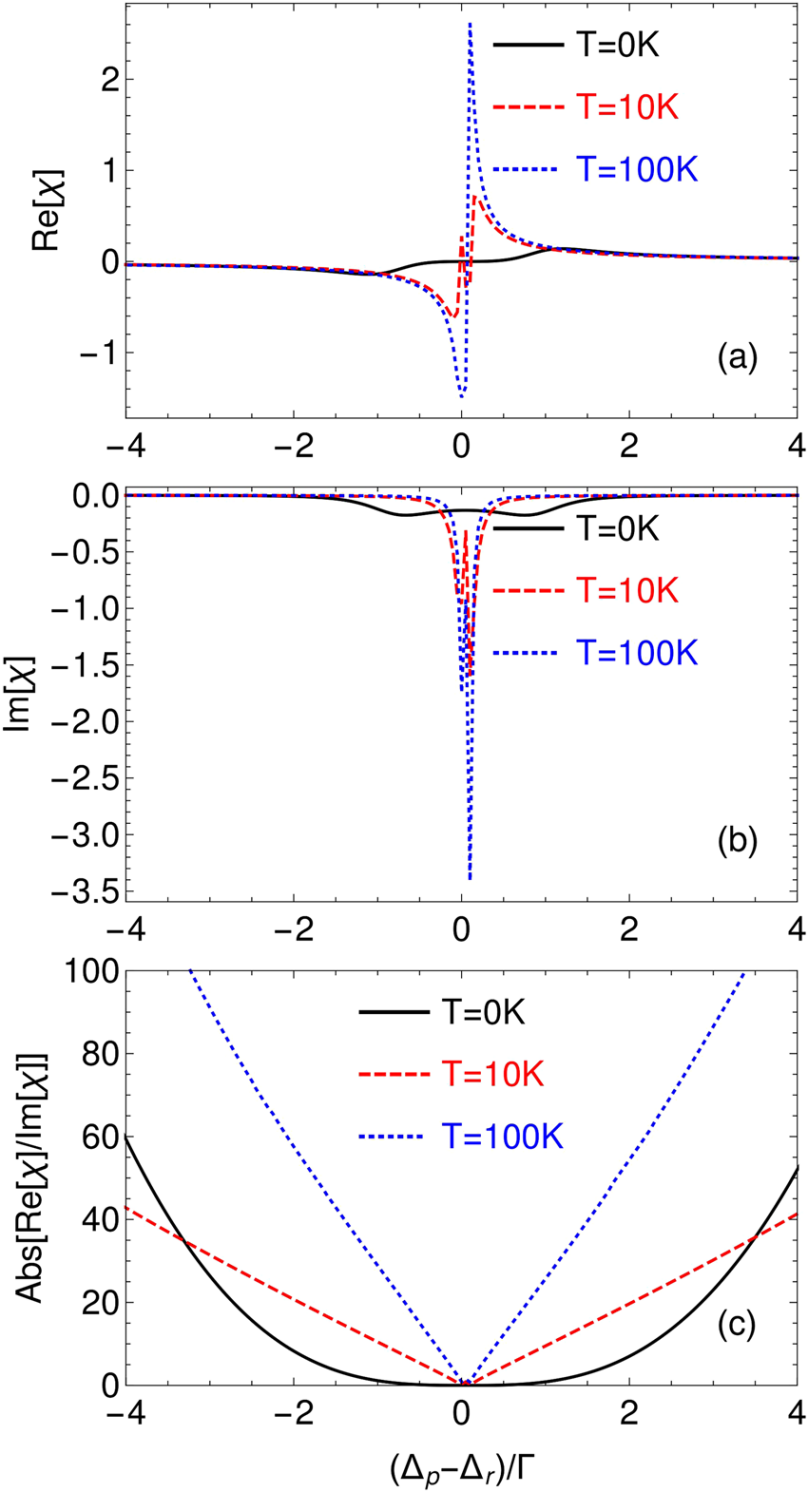
(**a**) Real and (**b**) imaginary parts of the linear susceptibility at three different temperatures $T=\mathrm{0\ }K,\mathrm{10\ }K,\mathrm{100\ }K$ respectively. It can be seen that the dispersion is enhanced while the gain is reduced in the “waveguide region” defined by Eq. (10), leading to better dispersion-gain contrast as shown in (**c**). The other parameters are the same as in Fig. 2.

In the previous studies, it has been shown that the linear susceptibility can be enhanced by the Doppler effect^29^. This is contrary to the region of single-photon resonance where the linear response of atoms is attenuated owing to Doppler broadening. We then wonder if the optical waveguide can be improved in the sense that the Doppler-averaged susceptibility can have better dispersion-gain contrast as given by Eq. (10b). In order to show this, we calculate the real and imaginary parts of the linear susceptibility at different temperatures *T* (see Fig. 4). The Doppler effect is included by substituting ${{\rm{\Delta }}}_{r},{{\rm{\Delta }}}_{p}$ and ${{\rm{\Delta }}}_{c}$ by ${{\rm{\Delta }}}_{r}-{k}_{r}v,{{\rm{\Delta }}}_{p}-{k}_{p}v$ and ${{\rm{\Delta }}}_{c}-{k}_{c}v$ respectively (${k}_{j}$ is the wavevector of the corresponding laser field and $v$ is the velocity of atoms). Then, we integrate over all velocities from $-\infty$ to $\infty$ for the linear susceptibility of the probe field

12 $$\chi ({\omega }_{p})=\frac{3{{\rm{\Gamma }}}_{32}}{8{\pi }^{2}}{\int }_{-\infty }^{\infty }\frac{N(v){\lambda }_{p}^{3}{\rho }_{32}^{\mathrm{(1)}}({\omega }_{p}\mathrm{.}v)}{{{\rm{\Omega }}}_{p}}dv,$$

where $N(v)={N}_{0}{e}^{-{v}^{2}/{v}_{p}^{2}}/(\sqrt{\pi }{v}_{p})$ is the Maxwell velocity distribution, and ${v}_{p}=\sqrt{2{k}_{B}T/m}$ is for the most probable velocity^31^. Indeed, owing to the Doppler effect, both the dispersion and gain are enhanced around the two-photon resonance as seen in Fig. 4(a) and (b). In particular, when the region of two-photon resonance enters into the “waveguide region” as defined by Eq. (10), the gain is considerably reduced. And if choosing the smaller two photon detuning ${{\rm{\Delta }}}_{p}-{{\rm{\Delta }}}_{r}$, the dispersion still remains strong, which can be obtained to induce the waveguide structure. The underlying reason is that there are always a group of moving atoms which match the resonance condition and contribute significantly to the linear responsibility as explained in more detail in Ref.^29^. This is more clearly demonstrated in Fig. (4c) that the dispersion-gain contrast is improved as *T* increases.

**Figure 5:**
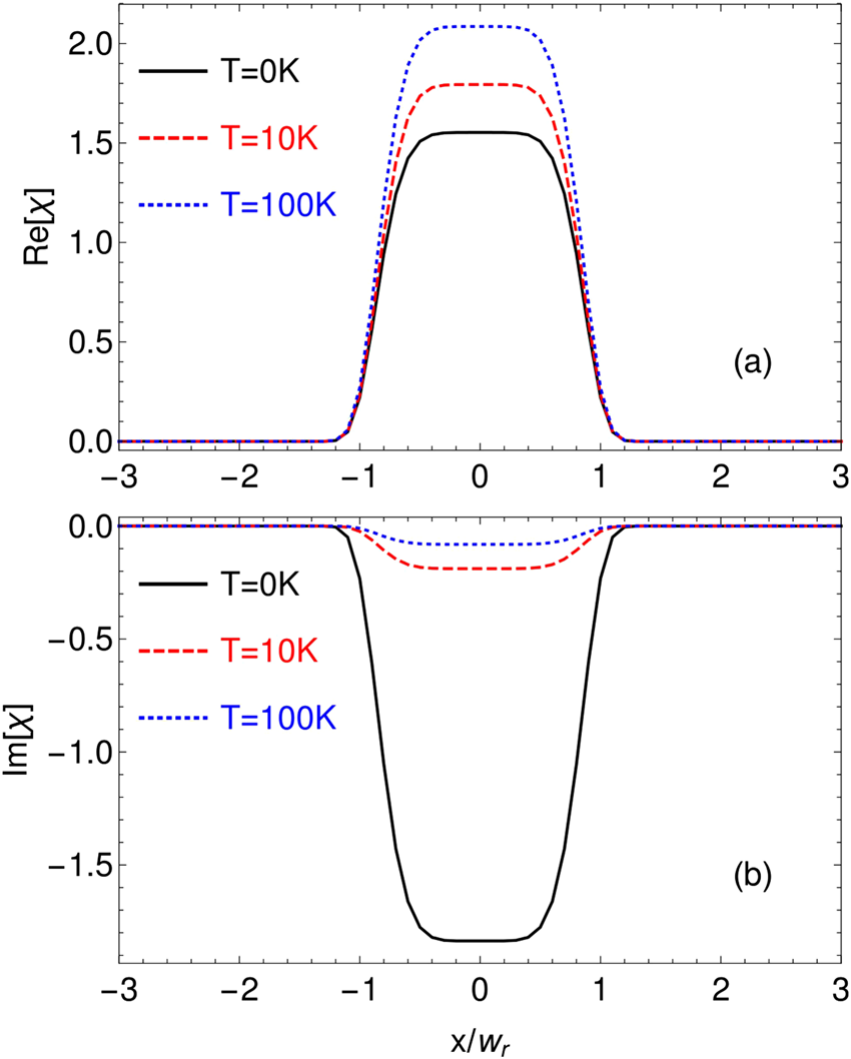
(**a**) Real and (**b**) imaginary parts of the linear susceptibility as a function of the position at different temperatures. It can be seen that the dispersion is enhanced by the Doppler effect while the gain is reduced, resulting in better waveguide effects. Here ${{\rm{\Delta }}}_{p}=201{\rm{\Gamma }}$ and other parameters are the same as in Fig. 2.

In the following, we then choose the probe detuning as ${{\rm{\Delta }}}_{p}\mathrm{=201}{\rm{\Gamma }}$ which corresponds to a better dispersion-gain contrast to study the induced waveguide structure. The linear susceptibility as a function of the position for different temperatures are shown in Fig. 5. It can be seen that the dispersion is enhanced while the gain is significantly reduced for higher temperature. It means that, better waveguide structures can be obtained by simply increasing the temperature, and the probe field would experience diffractionless and gain-free propagation.

## Propagation dynamics

In this section, we proceed to study the light propagation in our optically induced waveguide. In order to focus on the propagation dynamics of the probe field, we here assume that the beam size of the Raman field Ω_*r*_ remains unchanged during the probe propagation. For a first step, a Gaussian probe is chosen to propagate in the optically tailored medium to demonstrate its waveguide effect. The input of the probe has the form:

13 $${{\rm{\Omega }}}_{p}(x,y)={{\rm{\Omega }}}_{p0}{e}^{-\frac{{x}^{2}+{y}^{2}}{2{w}_{p}^{2}}},$$

where the amplitude ${{\rm{\Omega }}}_{p0}$ is much smaller than that of the control field, and the waist ${w}_{p}$ is chosen to be 100 *μm* which is much larger than its wavelength such that the paraxial approximation is safely retained.

**Figure 6:**
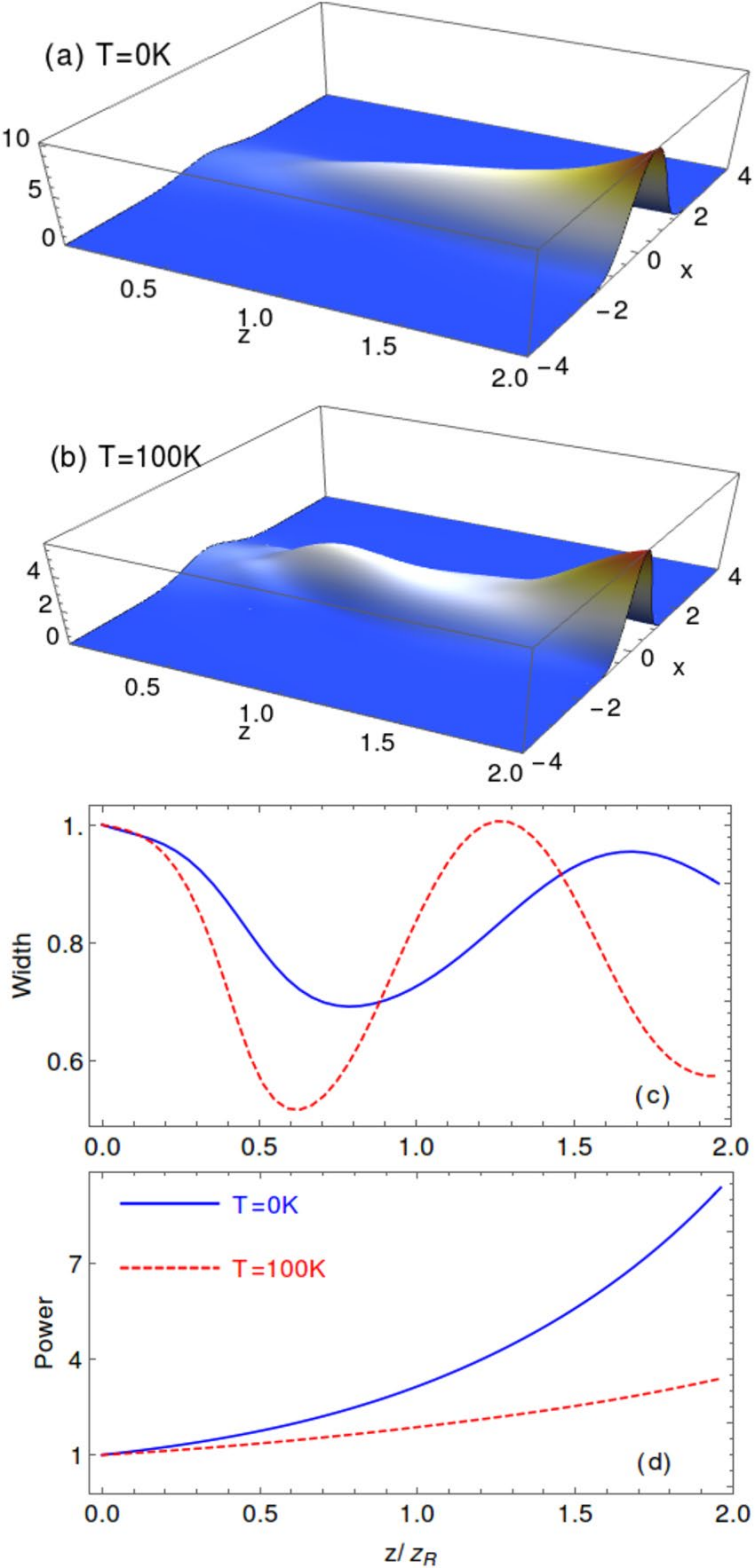
The propagation of the two-dimensional probe field in the prepared atomic medium with the active optical waveguide for two different temperatures (**a**) *T* = 0 *K* and (**b**) *T* = 100 *K*. The normalized width and power of the probe field as a function of propagation distance is calculated as in (**c**) and (**d**). Here we choose ${n}_{0}\mathrm{=5.0}\times {10}^{12}\,{{\rm{cm}}}^{-3}$, ${{\rm{\Delta }}}_{p}=202{\rm{\Gamma }}$ when *T* = 0 *K*, and ${\Delta }_{p}\mathrm{=201}{\rm{\Gamma }}$ when *T* = 100 *K*, other parameters are the same as in Fig. 2.

Owing to the choices of the control field and the incident probe fields which are spatially symmetric in the transverse plane, it is straightforward that the propagation dynamics should be also symmetric in the transverse degrees of freedom. We then reduce the 3D into 2D propagation as depicted in Fig. 6. Here the propagation distance is chosen to be two Rayleigh lengths $L=2{z}_{R}=4\pi {w}_{p}^{2}/{\lambda }_{p}\simeq \mathrm{15.8\ }cm$, and we have simulated the probe propagation in the optical prepared medium for two different temperatures as shown in Fig. 6(a) and (b). It is clear that in both cases the probe is mostly confined in the central region where the optically induced waveguide is formed as we have shown in the section above. Furthermore, as can be seen from Fig. 6(c), the width as a function of propagation distance shows oscillating behaviors which is just the phenomenon of total reflection as in the ray optics. Last but not the least, the power of the probe grows for increasing distance, which originates from the active nature of our optical waveguide.

When the Doppler effect is included, the width of the probe field oscillates faster against the propagation distance, and meanwhile the power growth slows down as shown in Fig. 6(b)–(d) with *T* = 100 *K*. It means that the induced optical waveguide works better at *T* = 100 *K* in the sense that the gain becomes less important. This is the direct consequence that the dispersion gets enhanced while the gain is weakened around the two-photon resonance by the Doppler effect as we discussed in the section above.

## Discussions and Conclusions

As can be seen from Fig. 5 that the Doppler effect can improve the optical waveguide in the sense that it reduces the Raman gain and enhances the dispersion, one may be interested if the optical waveguide can be refined by further increasing the temperature. However, the waveguide can not be unlimitedly improved by the Doppler effect. The reason that the gain is reduced by the Doppler effect comes from the fact that there is always a part of moving atoms being in single-photon resonance with the two fields and consequently introducing absorption. As the temperature grows further, this portion of moving atoms will become more important and eventually turns the Raman gain into absorption in the “waveguide region”. This suggests that there will be a transition point at certain temperature at which the gain is zero inside the induced waveguide, allowing the possibility of guiding light at few-photon level.

In the main text, we have not specify which atomic species should be used. In principle, any kind of atomic species which contains a four-level *N*-type structure would be suitable to realize our scheme. For example, one may choose ^87^Rb which is widely used in atomic physics laboratories for the experimental implementation. In fact, our numerical simulation are based on the parameters of the D1 line of ^87^Rb, i.e., ^5^S_1/2_, F = 1, 2 to be the lower states |1〉 and 2〉, ^5^P_1/2_, F = 1 to be state |3〉 and ^5^P_3/2_, F = 2 to be state |4〉.

In conclusion, we have proposed a scheme to induce an optical waveguide in a *N*-type four-level ^87^Rb atoms medium by using the ARG process. The resulting waveguide is active owing to the nature of ARG and can be further improved by the Doppler effect, in sharp contrast to the conventional optical waveguide-like structure which is usually accompanied with absorption and will be attenuated by the Doppler effect. We then studied the probe propagation dynamics in the optically tailored atomic medium which have shown a diffractionless and absorption-free propagation.
